# Exploring the Neural Representation of Novel Words Learned through Enactment in a Word Recognition Task

**DOI:** 10.3389/fpsyg.2016.00953

**Published:** 2016-06-28

**Authors:** Manuela Macedonia, Karsten Mueller

**Affiliations:** ^1^Information Engineering, Johannes Kepler University LinzLinz, Austria; ^2^Neural Mechanisms of Human Communication, Max Planck Institute for Human Cognitive and Brain SciencesLeipzig, Germany; ^3^Nuclear Magnetic Resonance Unit, Max Planck Institute for Human Cognitive and Brain SciencesLeipzig, Germany

**Keywords:** second language, word learning, enactment, embodiment, brain

## Abstract

Vocabulary learning in a second language is enhanced if learners enrich the learning experience with self-performed iconic gestures. This learning strategy is called enactment. Here we explore how enacted words are functionally represented in the brain and which brain regions contribute to enhance retention. After an enactment training lasting 4 days, participants performed a word recognition task in the functional Magnetic Resonance Imaging (fMRI) scanner. Data analysis suggests the participation of different and partially intertwined networks that are engaged in higher cognitive processes, i.e., enhanced attention and word recognition. Also, an experience-related network seems to map word representation. Besides core language regions, this latter network includes sensory and motor cortices, the basal ganglia, and the cerebellum. On the basis of its complexity and the involvement of the motor system, this sensorimotor network might explain superior retention for enactment.

## Introduction

In foreign language instruction, novel vocabulary is mainly taught by means of audio-visual strategies such as listening and comprehension activities (Graham et al., 2014). At home, learners are confronted with bilingual lists that decay fast (Yamamoto, 2014). Memory research has demonstrated that self-performed gestures accompanying words and phrases during learning enhance vocabulary retention compared to reading and/or listening (Zimmer, 2001). The effect of gestures on verbal memory, *enactment effect* (Engelkamp, 1980; Engelkamp and Krumnacker, 1980; Engelkamp and Zimmer, 1984) or *subject performed task effect* (Cohen, 1981), has proven to be robust. In the 1980s and 1990s, enactment of words and phrases was successfully tested in various populations, including children, young, and elderly people (Bäckman and Nilsson, 1985), subjects with cognitive and mental impairments (Mimura et al., 1998), and Alzheimer patients (Karlsson et al., 1989) by means of recognition and free and cued recall tests. In recent years, an increasing number of behavioral studies have documented the positive effect of enactment also in second language word learning in both the short and in the long term (for a review, see Macedonia, 2014). More recently, the combined value of enactment and physical exercise has been investigated in elementary bilingual instruction (Mavilidi et al., 2015; Toumpaniari et al., 2015).

Over the decades, different theories have accounted for the enactment effect. Nearly 40 years ago, these theories based their evidence on behavioral experiments and were nourished by observation and a large portion of intuition. With the advent of neuroscience, these theories have gained additional empirical ground. They have been partially validated through combined experiments employing tools from both disciplines, i.e., behavioral psychology and brain imaging as we describe in the next sections.

The first theory asserts that a gesture performed by learners during the acquisition of a novel word leaves a motor trace in memory (Engelkamp and Krumnacker, 1980; Engelkamp and Zimmer, 1984, 1985; Nyberg et al., 2001). In neuroscience, a *trace* is an experience-related component in the functional network representing the word (Pulvermüller, 2002); neuroimaging studies have proven the existence of the trace. During acoustic and audio-visual recognition of words learned through enactment, motor cortices become active (Nyberg et al., 2001; Masumoto et al., 2006; Eschen et al., 2007; Macedonia et al., 2011; Mayer et al., 2015). More generally, this theory can be embedded in the framework of embodied cognition (Barsalou, 2008), where a concept entails sensory, motor and/or emotive components pertaining to the corresponding sensory, motor and/or affective systems engaged during experience (Jirak et al., 2010). The trace can be detected by means of brain imaging that locates brain activity during word recognition/retrieval. This activity is due to simulation processes that reactivate neural ensembles originally involved and interconnected during the experience (Dijkstra and Post, 2015).

Another theory attributes the enactment effect to *mental imagery*. Saltz and Donnenwerthnolan (1981) suggested that performing a gesture to a word triggers the mental image associated with the word. Thereafter the double representation (verbal and visual) enhances the word's retention. This view is connected to Paivio's dual-coding theory (Paivio, 1969, 1971), which maintains that an image paired to a word has an impact on the word's retention because the processing of both word and image engages different channels and exploits both potentials. Imagery related to L2 word learning has also been tested in a brain imaging experiment: Macedonia et al. (2011) had subjects learn L2 words with two sets of gestures, iconic and semantically unrelated. Audiovisual presentation of words learned with semantically unrelated gestures elicited activity in a network engaged in cognitive control. These results were interpreted as detection of the mismatch between the mental image a person has of a word and the gesture used during training. In other studies, mismatches between L1 words and gestures were seen as activating a network denoting incongruence (Holle and Gunter, 2007; Holle et al., 2008). Altogether, in recent decades a growing body of evidence has demonstrated that language and gesture represent two aspects of the same communicative system (Goldin-Meadow, 1999; Bernardis and Gentilucci, 2006; Kelly et al., 2010) and that they share neural substrates (for a review, see Andric and Small, 2012).

In a few behavioral studies, the enactment effect has also been explained in terms of *complexity* of word representation (Knopf, 1992; Kormi-Nouri, 1995; Macedonia, 2003; Macedonia and Knösche, 2011). In these studies, the term complexity was used in a descriptive way: The authors asserted that a written word enriched by a gesture is represented in memory in a more complex way because additional perceptive modalities are engaged. Neuroscience has demonstrated that the brain codes, represents and stores information connected to a word on the basis of the sensory input provided (Pulvermüller, 2001, 2005; Pulvermüller and Fadiga, 2010). Hence gestures make the word's representation richer and more complex. In traditional L2 instruction, learning a written word or listening to it leads to a representation that is bare of multiple sensorimotor experiences as in L1. Gestures as a learning strategy add sensorimotor and proprioceptive information to the word in L2. Thereby the word's representation becomes more complex and elaborative.

A further explanation for the enactment effect focuses on *enhanced attention*. Supporters of this theory assert that learners performing a gesture connected to a word's semantics are more attentive than those who only read verbal information or hear the word (Backman et al., 1993; Knudsen, 2007; Muzzio et al., 2009a,b; Pereira et al., 2012). Attention is a basic component of retention as it induces representational stability in the hippocampus (Muzzio et al., 2009a; Aly and Turk-Browne, 2016).

The superior retrievability of L2 words learned through enactment has also been accounted for in terms of depth of encoding (Quinn-Allen, 1995; Macedonia, 2003; Tellier, 2008; Kelly et al., 2009; Macedonia et al., 2011; Krönke et al., 2013). The concept of deep and shallow encoding goes back to the “Level of Processing Framework” (LOP) by Craik and Tulving (1975). In LOP, sensory processing—hearing a word—is shallow information encoding that leads to poor memory performance (Craik and Lockhart, 1972). By contrast, semantic processing by selecting semantic features of a word in a task is deep and leads to durable memorization (Hunt and Worthen, 2006). Furthermore, deep processing is achieved by integrating novel information with pre-existing knowledge and creating distinctiveness during encoding. Thus, deep processing could be accomplished through gestures. Performing a gesture means selecting arbitrary features related to a word that represent its semantics. Furthermore, gestures can integrate novel information, i.e., the word in L2, with pre-existing semantic knowledge about the word in L1. Although depth of processing has been taken as an explanation for successful memorization in many studies on enactment, the brain mechanisms associated with depth are not fully understood. In an early review article, Nyberg (2002) connected deep processing with brain activity in frontal and medial temporal brain regions. More recently, Galli (2014) reviews functional magnetic resonance imaging (fMRI) studies on tasks with shallow vs. deep encoding; the author concludes that brain regions engaged in shallow encoding represent a subset of those involved in deep encoding. Galli concludes that shallow and deep encoding might have varying network topographies depending on the kind of stimulus processed and the specificity of the encoding tasks.

While early enactment research attributed the effect to only one of the above reasons, recent research indicates that the different accounts mirror different aspects of enacting verbal information. A gesture accompanying a novel word creates a motor trace, triggers a mental image, encodes more deeply, and obviously engages more attention than only reading the word. All these processes are performed in networks interacting in brain topography and time.

Another theory on enactment, the *system-oriented approach* pursued by Engelkamp (2001), has not received much attention in the scientific discourse. Following on the line of memory subsystems (Engelkamp and Zimmer, 1994), Engelkamp advances the hypothesis that enactment, because of the “physical properties of the ongoing stimulus” might engage “more than one memory system.” More explicitly, encoding a written word as such (a character string) involves *explicit*, i.e., declarative memory. However, encoding a gesture, an *action*, stores the information related to the word in procedural memory. Accordingly, because enactment of verbal information engages “systems that are obligatorily activated dependently on the stimulus modality” combines both declarative and procedural memory during learning. Engelkamp observes that “there are systems that are not automatically activated given a specific stimulus, but that can be strategically activated, for instance, when a specific task is given.” In other words, the procedural system is not automatically engaged when we learn words by reading them or listening to them. However, the procedural system can be strategically involved by accompanying the words with gestures. Gestures therefore lead to memory enhancement because—by the nature of the stimulus—they engage procedural memory in word storage. Daprati et al. (2005) also address the possible involvement of procedural memory for words encoded by enactment. The study is conducted on healthy individuals and on schizophrenic patients. The latter show deficits in awareness states but procedural memory is intact (Danion et al., 2001). Healthy subjects take advantage of the enactment effect but patients suffering from schizophrenia do not. This result does not confirm the engagement of procedural memory in learning through enactment; however, schizophrenia has been reported to be a motor awareness disturbance (Frith et al., 2000) with deficits in monitoring of self-generated actions (Frith, 1987). This might explain the failure of enactment in this specific population.

Despite the progress in enactment research, we still lack a complete picture of how novel words learned with iconic gestures are functionally mapped in the brain. The present study aims to show the different experience-related components of the word network. To this end, we use a section of a fMRI dataset originally acquired to investigate differences in retention between words learned with iconic gestures and words learned with semantically unrelated gestures (Macedonia et al., 2011). We designed that study in order to test word retention learned with iconic and semantically unrelated gestures. At that time, it was not clear which component was crucial to retention: the motor component or iconicity. We found that words accompanied by iconic gestures were better retained than words learned with semantically unrelated gestures and that this correlated with stronger activity in the motor cortices. Furthermore, words learned with semantically unrelated gestures elicited a network for cognitive control, possibly denoting a mismatch between an internal image of the word and the gesture presented while learning.

In the present study, we extract from the dataset those events that are related to the recognition of words learned with iconic gestures. By analyzing the BOLD response elicited during recognition of the L2 words, we seek to localize networks involved in learning. We hypothesize that these networks include the sensorimotor modalities engaged during the process. Further, we follow Engelkamp's system-oriented approach and pose the question of whether other brain structures related to procedural memory—besides motor cortices, as demonstrated in previous studies—may be involved in a word's representation. If so, this could provide evidence for the engagement of procedural memory in word learning through enactment.

## Methods

### Participants, behavioral training procedure, and results

**Figure 1:**
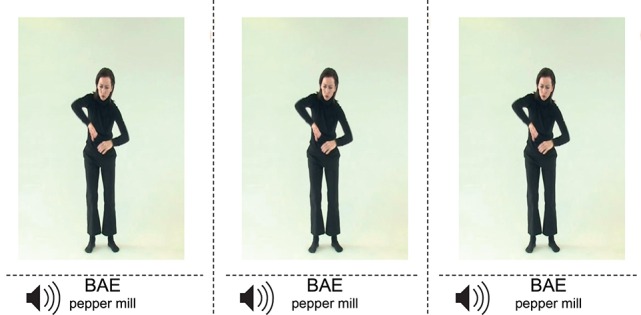
**Screen shot of an iconic gesture from the video used during training**. It represents the Vimmi word bae (Engl. pepper mill). During the training, participants were cued to perform the gesture as they said the word after reading and hearing it.

Eighteen participants (mean age 23.44, M = 25, SD = 1.38, 10 females, 8 males) memorized 92 words of Vimmi, an artificial corpus created in order to avoid associations to languages known to the subjects and conforming to Italian phonotactic rules (Macedonia et al., 2011). During the training, lasting for 4 days, 2 h daily, the novel words were accompanied either by 46 iconic (McNeill, 1992) or by 46 semantically unrelated gestures such as stretching one's arms. Participants watched videos of an actress performing the gestures and enunciating the novel words (Figure 1). Simultaneously, the written word in Vimmi and in German appeared on a screen. Thereafter, participants were cued to repeat the word in Vimmi and to perform the corresponding gesture. Participants were randomly divided in two subgroups that trained both sets of words and gestures in a counterbalanced way. The 92 words were subdivided in blocks of 6 + 6 + 6 + 5 items. Within each block, every vocabulary item was randomized and presented daily 13 times. From the second until the fifth experiment day, memory performance was assessed through cued translation tests from German into Vimmi and vice versa. Starting from day 2, before the training participants were given a randomized list of the 92 trained words to be translated into the other language (duration 7.5 min for every list). Additionally the same test was administered after ~60 days.

**Figure 2:**
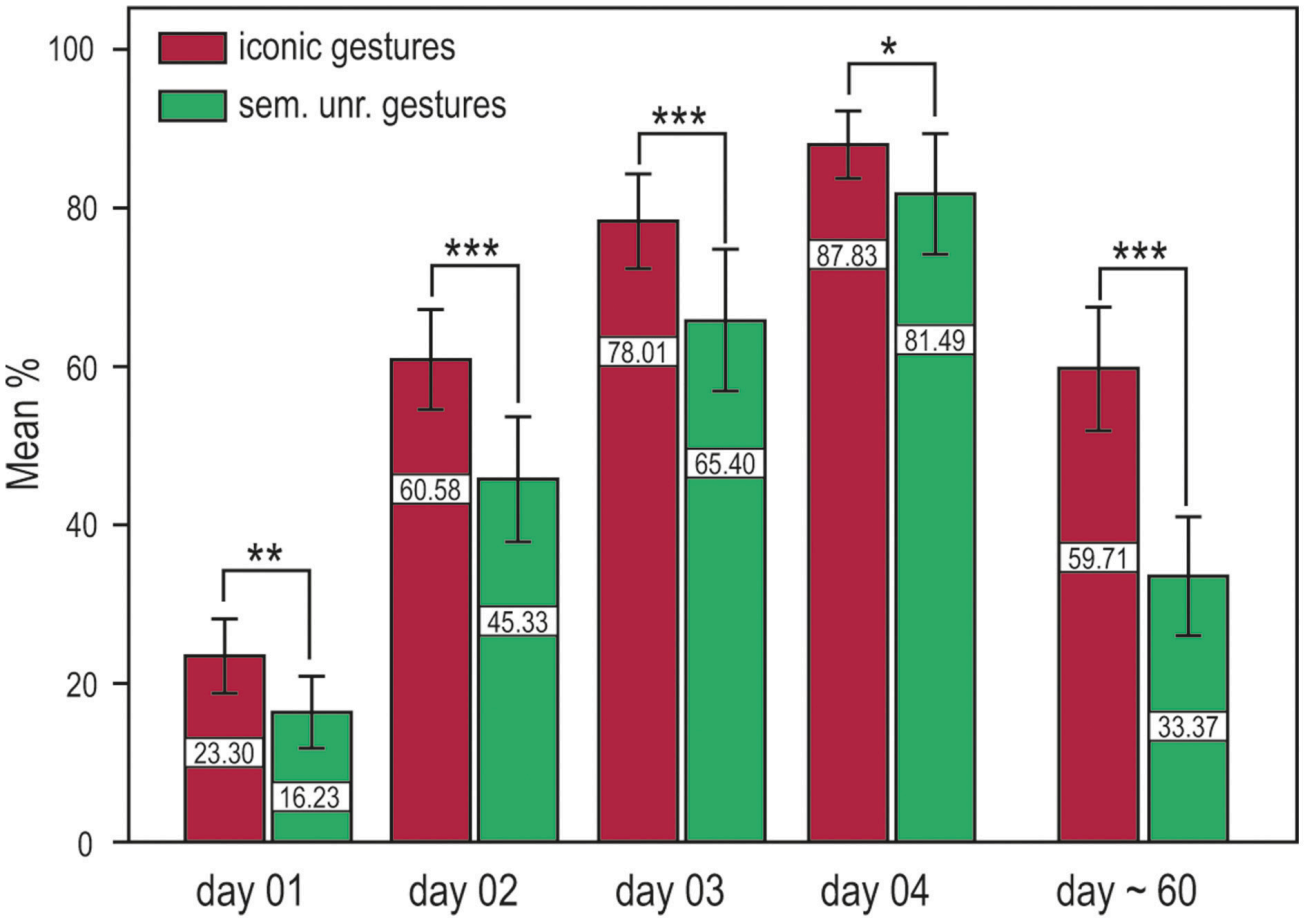
**Training results for the cued translation test from German into Vimmi and vice versa (merged data) and results for the paired free recall test (day 60)**. Words encoded through iconic gestures are significantly better retrieved at all time points. ^*^p < 0.05, ^**^p < 0.01, ^***^p < 0.001.

We performed a repeated measures ANOVA with the factors training and time (iconic and semantically unrelated gestures) and time (DAY 01, DAY 02, DAY 03, DAY 04). Significantly better results in word retention were achieved with iconic gestures in both the short and long term. For both translation directions, memory performance was significantly better for words learned with iconic gestures, i.e., German into Vimmi = Training *F*_(1, 32)_ = 22.86, *p* < 0.001 and Vimmi into German = *F*_(1, 32)_ = 15.20, *p* < 0.001. Additionally, around 60 days after training, memory performance was tested again by means of a paired free recall test. Participants were first given an empty sheet and were asked to write down as many words as they could remember in either one or the other language with the corresponding translation. This test mirrors the capacity of a second language learner to retrieve the word in both languages. The free recall test showed superior memory results for words learned with iconic gestures *F*_(1, 28)_ = 122.18, *p* < 0.001 (Figure 2).

### fMRI experiment

**Figure 3:**
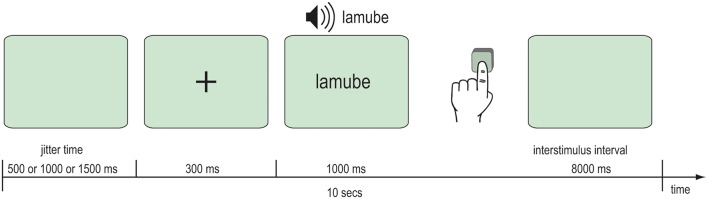
**Scanning procedure**.

After the behavioral learning phase, fMRI data were acquired during an audio-visual word recognition task. In the scanner, participants were audio-visually presented 92 words that they had previously trained and 23 novel words. Further 23 silent events represented the baseline. A single item was presented at each trial beginning with a fixation cross for 300 ms followed by the Vimmi word for 1 s. The interstimulus interval was 8 s. Participants read the words on a back-projection screen mounted behind their heads in the bore of the magnet. Audio files with an approximate duration of 1000 ms were played when the word was shown. Participants were instructed to press a key with their left hand if the word was unknown. Altogether the scanning comprised 138 trials with the trained and the novel words and the silent events. All items were balanced across the presentation. The whole experimental session lasted 23 min (Figure 3).

### Neuroimaging parameters

Functional scanning was performed with the following imaging parameters: BOLD sensitive gradient EPI sequence, TR = 2000 ms, TE = 30 ms, flip angle = 90°, acquisition bandwidth = 100 Hz. We acquired 20 axial slices (4 mm thick, 1 mm interslice distance, FOV 19.2 cm, data matrix of 64 × 64 voxels, inplane resolution of 3 × 3 mm) every 2000 ms on a 3-T Bruker (Ettlingen, Germany) Medspec 30/100 system. Prior to functional data acquisition, we obtained a T1-weighted modified driven equilibrium Fourier transform (MDEFT) image (data matrix 256 × 256, TR = 130 ms, TE = 10 ms) with a non-slice-selective inversion pulse that was followed by a single excitation of each slice (Norris, 2000). This anatomical image (which was acquired in the same orientation as the functional images) was co-registered with a previously obtained high-resolution whole-head 3-D brain image: 128 sagittal slices, 1.5-mm thickness, FOV 25.0 × 25.0 × 19.2 cm, data matrix of 256 × 156 voxels. Thereafter, the same registration parameters were applied to the functional images. The fMRI experiment was approved by the Ethics Committee of the University of Leipzig (Germany).

### Data analysis

For the present paper, we considered only the data related to the words learned through iconic gestures and contrasted them with the baseline silence. We intended to explore the topography of experience-related word networks. We analyzed the functional data with the Lipsia software package (Lohmann et al., 2001). Functional data were corrected for motion and for the temporal offset between the slices. Thereafter, we aligned the functional slices with a 3D stereotactic coordinate reference system. We acquired the registration parameters on the basis of the MDEFT slices, thereby achieving an optimal match between the slices and the individual 3D reference dataset, standardized to the Talairach stereotactic space (Talairach and Tournoux, 1988). We transformed the functional slices using trilinear interpolation, so that the resulting functional slices were aligned with the stereotactic coordinate system according to the registration parameters. During pre-processing, we further smoothed the data with a Gaussian filter of 10 mm FWHM, and a temporal high-pass filter with a cut-off frequency of 1/100 Hz. The data were entered as statistics using general linear regression with pre-whitening (Worsley et al., 2002). By using the Yule–Walker equations from the least squares residuals, we estimated autocorrelation parameters. They were also used to whiten both data and design matrices. Thereafter, we re-estimated the linear model using least squares on the whitened data to produce estimates of effects and their standard errors. We generated the design matrix using the canonical hemodynamic response function (Friston et al., 1998). Subsequently, we generated contrast images by computing the difference between the parameter estimates of the iconic gestures condition and the baseline, i.e., silence. We entered all contrast images into a second-level Bayesian analysis. Compared with null hypothesis significance, this analysis has a high reliability in small-group statistics with high within-subject variability (Friston et al., 2008). For display reasons, Bayesian probabilities (1-*p*) were finally transformed to *z*-values.

### fMRI results

**Figure 4:**
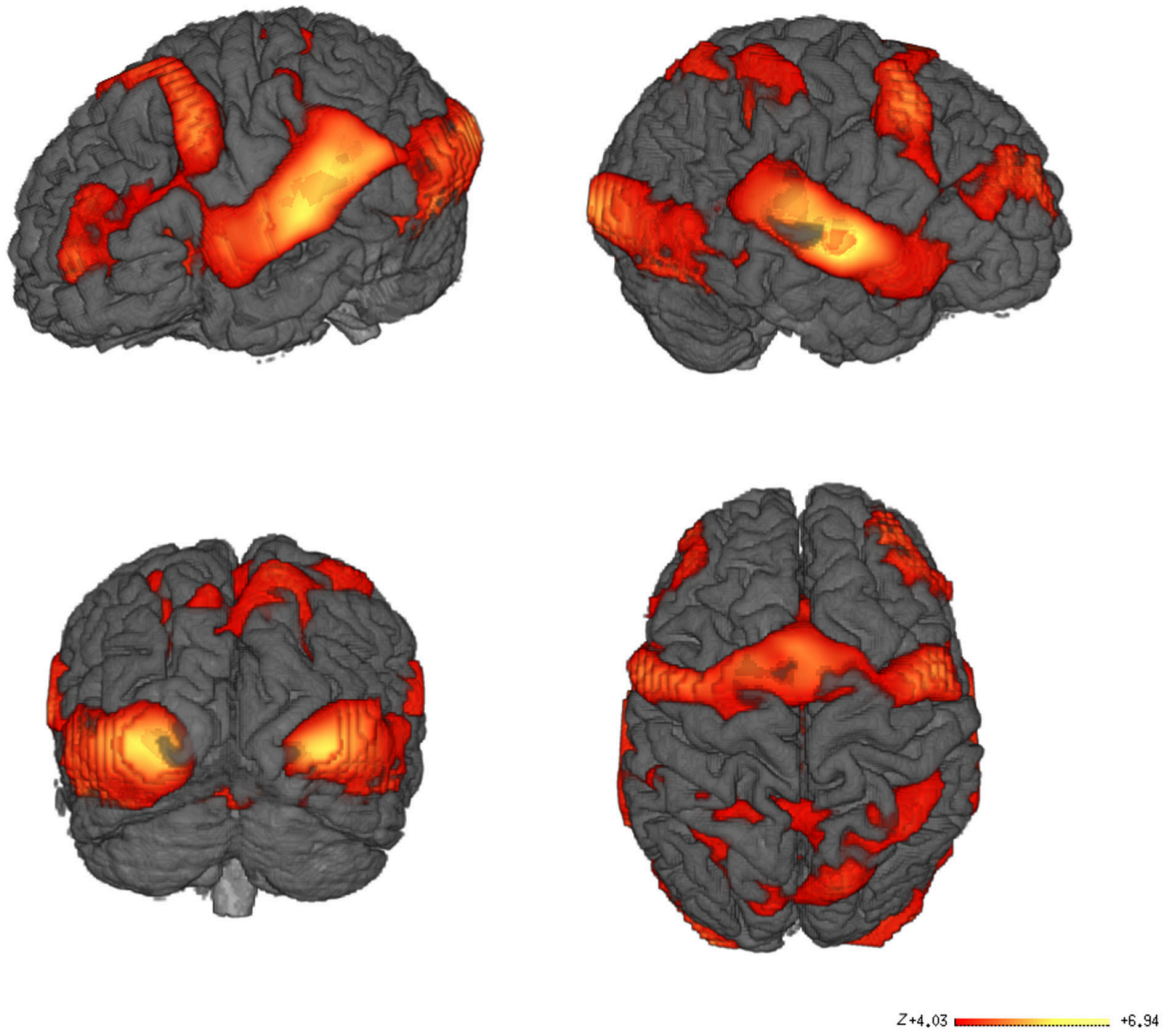
**fMRI Results**. Main contrast for words learned with iconic gestures vs. silence. Learning through iconic gestures creates extended sensorimotor networks that resonate upon audio-visual word presentation. The networks map the modalities engaged during learning. The color-coded areas show clusters with high Bayesian posterior probability of condition. The bar represents the *z*-values.

Our data were acquired during a word recognition task when participants lying in the scanner were visually and acoustically presented words that they had previously learned with iconic gestures, and unknown words, in random order. The whole brain analysis of the contrast between all words learned with iconic gestures vs. the baseline silence revealed haemodynamic responses in a number of regions as listed in Table 1. As fMRI lacks temporal resolution for neural processes, our visualization of regional oxygenated blood flow seems to reflect processes of perception, attention and word selection that occurred during scanning, as well as the functional neural representation of the words (Figure 4).

**Table 1:** **Results of fMRI main contrast (Silence—Iconic Gestures)**.

**CEREBRUM Talairach**				
**x**	**y**	**z**	***z*-val**	
**BA 3**				
27	−33	60	3.965	Right Cerebrum, Parietal Lobe, Postcentral Gyrus, Gray Matter, Brodmann area 3, Range = 1
21	−36	60	4.031	Right Cerebrum, Parietal Lobe, Postcentral Gyrus, Gray Matter, Brodmann area 3, Range = 1
**BA 4**				
−63	−21	42	3.068	Left Cerebrum, Frontal Lobe, Precentral Gyrus, Gray Matter, Brodmann area 4, Range = 3
**BA 5**				
−33	−39	60	4.205	Left Cerebrum, Parietal Lobe, Postcentral Gyrus, Gray Matter, Brodmann area 5, Range = 0
**BA 6**				
−21	3	66	5.123	Left Cerebrum, Frontal Lobe, Superior Frontal Gyrus, Gray Matter, Brodmann area 6, Range = 0
−54	0	39	5.526	Left Cerebrum, Frontal Lobe, Precentral Gyrus, Gray Matter, Brodmann area 6, Range = 0
−18	3	48	5.583	Left Cerebrum, Frontal Lobe, Medial Frontal Gyrus, Gray Matter, Brodmann area 6, Range = 1
−45	0	57	5.140	Left Cerebrum, Frontal Lobe, Middle Frontal Gyrus, Gray Matter, Brodmann area 6, Range = 2
−36	−9	36	5.131	Left Cerebrum, Frontal Lobe, Precentral Gyrus, Gray Matter, Brodmann area 6, Range = 2
−42	−3	30	5.532	Left Cerebrum, Frontal Lobe, Precentral Gyrus, Gray Matter, Brodmann area 6, Range = 2
−39	−3	60	5.192	Left Cerebrum, Frontal Lobe, Middle Frontal Gyrus, Gray Matter, Brodmann area 6, Range = 3
−30	0	39	5.213	Left Cerebrum, Frontal Lobe, Middle Frontal Gyrus, Gray Matter, Brodmann area 6, Range = 4
−21	−9	39	5.349	Left Cerebrum, Frontal Lobe, Middle Frontal Gyrus, Gray Matter, Brodmann area 6, Range = 4
−30	−12	39	5.110	Left Cerebrum, Frontal Lobe, Middle Frontal Gyrus, Gray Matter, Brodmann area 6, Range = 4
36	−3	54	5.751	Right Cerebrum, Frontal Lobe, Middle Frontal Gyrus, Gray Matter, Brodmann area 6, Range = 0
**BA 7**				
−30	−54	42	6.615	Left Cerebrum, Parietal Lobe, Superior Parietal Lobule, Gray Matter, Brodmann area 7, Range = 3
−12	−45	48	4.749	Left Cerebrum, Parietal Lobe, Precuneus, Gray Matter, Brodmann area 7, Range = 1
−18	−69	39	5.020	Left Cerebrum, Parietal Lobe, Precuneus, Gray Matter, Brodmann area 7, Range = 0
27	−48	57	4.834	Right Cerebrum, Parietal Lobe, Superior Parietal Lobule, Gray Matter, Brodmann area 7, Range = 1
12	−66	51	5.507	Right Cerebrum, Parietal Lobe, Precuneus, Gray Matter, Brodmann area 7, Range = 1
18	−45	45	5.296	Right Cerebrum, Parietal Lobe, Precuneus, Gray Matter, Brodmann area 7, Range = 2
24	−54	39	6.106	Right Cerebrum, Parietal Lobe, Precuneus, Gray Matter, Brodmann area 7, Range = 5
**BA 8**				
39	21	48	2.415	Right Cerebrum, Frontal Lobe, Superior Frontal Gyrus, Gray Matter, Brodmann area 8, Range = 0
**BA 9**				
−42	39	33	4.744	Left Cerebrum, Frontal Lobe, Superior Frontal Gyrus, Gray Matter, Brodmann area 9, Range = 0
−54	27	33	4.281	Left Cerebrum, Frontal Lobe, Middle Frontal Gyrus, Gray Matter, Brodmann area 9, Range = 0
0	15	33	6.229	Left Cerebrum, Frontal Lobe, Cingulate Gyrus, Gray Matter, Brodmann area 32, Range = 2
−12	−18	33	5.317	Left Cerebrum, Limbic Lobe, Cingulate Gyrus, Gray Matter, Brodmann area 24, Range = 5
−48	12	24	4.619	Left Cerebrum, Frontal Lobe, Inferior Frontal Gyrus, Gray Matter, Brodmann area 9, Range = 2
27	48	33	5.256	Right Cerebrum, Frontal Lobe, Superior Frontal Gyrus, Gray Matter, Brodmann area 9, Range = 1
30	33	24	5.629	Right Cerebrum, Frontal Lobe, Middle Frontal Gyrus, Gray Matter, Brodmann area 9, Range = 2
**BA 10**				
30	48	27	5.246	Right Cerebrum, Frontal Lobe, Superior Frontal Gyrus, Gray Matter, Brodmann area 10, Range = 2
30	57	18	4.980	Right Cerebrum, Frontal Lobe, Middle Frontal Gyrus, Gray Matter, Brodmann area 10, Range = 1
27	42	12	5.109	Right Cerebrum, Frontal Lobe, Middle Frontal Gyrus, Gray Matter, Brodmann area 10, Range = 5
**BA 11**				
−30	39	−12	2.876	Left Cerebrum, Frontal Lobe, Middle Frontal Gyrus, Gray Matter, Brodmann area 11, Range = 0
**BA 13**				
−30	6	21	5.532	Left Cerebrum, Sub-lobar, Insula, Gray Matter, Brodmann area 13, Range = 4
−30	21	6	6.619	Left Cerebrum, Sub-lobar, Insula, Gray Matter, Brodmann area 13, Range = 1
30	12	18	5.846	Right Cerebrum, Sub-lobar, Insula, Gray Matter, Brodmann area 13, Range = 5
54	−33	18	5.989	Right Cerebrum, Sub-lobar, Insula, Gray Matter, Brodmann area 13, Range = 1
**BA 18**				
−24	−96	0	6.494	Left Cerebrum, Occipital Lobe, Cuneus, Gray Matter, Brodmann area 18, Range = 1
15	−75	18	3.324	Right Cerebrum, Occipital Lobe, Cuneus, Gray Matter, Brodmann area 18, Range = 1
24	−93	0	6.618	Right Cerebrum, Occipital Lobe, Cuneus, Gray Matter, Brodmann area 18, Range = 2
**BA 20**				
−39	−12	−18	4.996	Left Cerebrum, Temporal Lobe, Sub-Gyral, Gray Matter, Brodmann area 20, Range = 1
**BA 22**				
−66	−48	12	5.722	Left Cerebrum, Temporal Lobe, Superior Temporal Gyrus, Gray Matter, Brodmann area 22, Range = 0
**BA 23**				
−3	−15	27	5.883	Left Cerebrum, Limbic Lobe, Cingulate Gyrus, Gray Matter, Brodmann area 23, Range = 0
0	−33	24	5.374	Left Cerebrum, Limbic Lobe, Cingulate Gyrus, Gray Matter, Brodmann area 23, Range = 2
**BA 24**				
18	−9	42	5.158	Right Cerebrum, Limbic Lobe, Cingulate Gyrus, Gray Matter, Brodmann area 24, Range = 3
15	−3	39	5.046	Right Cerebrum, Limbic Lobe, Cingulate Gyrus, Gray Matter, Brodmann area 24, Range = 4
3	3	30	5.225	Right Cerebrum, Limbic Lobe, Cingulate Gyrus, Gray Matter, Brodmann area 24, Range = 0
**BA 25**				
−9	15	−15	4.249	Left Cerebrum, Frontal Lobe, Medial Frontal Gyrus, Gray Matter, Brodmann area 25, Range = 1
**BA 27**				
9	−36	3	4.960	Right Cerebrum, Limbic Lobe, Parahippocampal Gyrus, Gray Matter, Brodmann area 27, Range = 2
**BA 29**				
−9	−39	12	4.868	Left Cerebrum, Limbic Lobe, Posterior Cingulate, Gray Matter, Brodmann area 29, Range = 4
**BA 31**				
12	−33	42	4.746	Right Cerebrum, Limbic Lobe, Cingulate Gyrus, Gray Matter, Brodmann area 31, Range = 3
**BA 32**				
18	33	18	5.318	Right Cerebrum, Limbic Lobe, Anterior Cingulate, Gray Matter, Brodmann area 32, Range = 0
**BA 36**				
−24	−33	−15	4.128	Left Cerebrum, Limbic Lobe, Parahippocampal Gyrus, Gray Matter, Brodmann area 36, Range = 1
−36	−30	−21	4.687	Left Cerebrum, Limbic Lobe, Parahippocampal Gyrus, Gray Matter, Brodmann area 36, Range = 0
**BA 37**				
−51	−72	0	5.590	Left Cerebrum, Occipital Lobe, Inferior Temporal Gyrus, Gray Matter, Brodmann area 37, Range = 0
−42	−63	−12	6.291	Left Cerebrum, Temporal Lobe, Fusiform Gyrus, Gray Matter, Brodmann area 37, Range = 0
42	−57	−18	5.604	Right Cerebrum, Temporal Lobe, Fusiform Gyrus, Gray Matter, Brodmann area 37, Range = 0
42	−42	−6	5.503	Right Cerebrum, Temporal Lobe, Fusiform Gyrus, Gray Matter, Brodmann area 37, Range = 5
**BA 38**				
−30	9	−21	3.815	Left Cerebrum, Temporal Lobe, Superior Temporal Gyrus, Gray Matter, Brodmann area 38, Range = 2
−54	21	−21	3.566	Left Cerebrum, Temporal Lobe, Superior Temporal Gyrus, Gray Matter, Brodmann area 38, Range = 2
**BA 39**				
−54	−69	36	−3.767	Left Cerebrum, Parietal Lobe, Angular Gyrus, Gray Matter, Brodmann area 39, Range = 2
**BA 40**				
−57	−33	30	4.984	Left Cerebrum, Parietal Lobe, Inferior Parietal Lobule, Gray Matter, Brodmann area 40, Range = 1
**BA 41**				
−54	−18	6	6.565	Left Cerebrum, Temporal Lobe, Superior Temporal Gyrus, Gray Matter, Brodmann area 41, Range = 0
36	−27	12	6.112	Right Cerebrum, Temporal Lobe, Transverse Temporal Gyrus, Gray Matter, Brodmann area 41, Range = 0
60	−18	6	7.005	Right Cerebrum, Temporal Lobe, Superior Temporal Gyrus, Gray Matter, Brodmann area 41, Range = 3
**BA 42**				
−63	−30	9	6.330	Left Cerebrum, Temporal Lobe, Superior Temporal Gyrus, Gray Matter, Brodmann area 42, Range = 0
**BA 44**				
−57	6	6	5.390	Left Cerebrum, Frontal Lobe, Precentral Gyrus, Gray Matter, Brodmann area 44, Range = 1
**BA 46**				
−39	48	15	5.116	Left Cerebrum, Frontal Lobe, Middle Frontal Gyrus, Gray Matter, Brodmann area 46, Range = 1
−60	30	9	2.669	Left Cerebrum, Frontal Lobe, Inferior Frontal Gyrus, Gray Matter, Brodmann area 46, Range = 2
**BA 47**				
−33	27	−18	2.982	Left Cerebrum, Frontal Lobe, Inferior Frontal Gyrus, Gray Matter, Brodmann area 47, Range = 1
33	18	0	6.942	Right Cerebrum, Sub-lobar, Extra-Nuclear, Gray Matter, Brodmann area 47, Range = 1
18	30	−15	3.595	Right Cerebrum, Frontal Lobe, Inferior Frontal Gyrus, Gray Matter, Brodmann area 47, Range = 2
**SUBCORTICAL STRUCTURES**				
**Thalamus**				
−21	−33	3	5.011	Left Cerebrum, Sub-lobar, Thalamus, Gray Matter, Pulvinar, Range = 0
−18	−12	9	5.434	Left Cerebrum, Sub-lobar, Thalamus, Gray Matter, Ventral Lateral Nucleus, Range = 1
−12	−18	9	5.298	Left Cerebrum, Sub-lobar, Thalamus, Gray Matter, Range = 0
9	−24	21	4.967	Right Cerebrum, Sub-lobar, Thalamus, Gray Matter, Range = 4
6	−12	9	5.594	Right Cerebrum, Sub-lobar, Thalamus, Gray Matter, Medial Dorsal Nucleus, Range = 0
**Caudate**				
−18	27	9	4.207	Left Cerebrum, Sub-lobar, Caudate, Gray Matter, Caudate Head, Range = 5
21	9	18	6.066	Right Cerebrum, Sub-lobar, Caudate, Gray Matter, Caudate Body, Range = 5
9	0	9	6.425	Right Cerebrum, Sub-lobar, Caudate, Gray Matter, Caudate Body, Range = 1
**Hippocampus**				
33	−12	−15	5.527	Right Cerebrum, Limbic Lobe, Parahippocampal Gyrus, Gray Matter, Hippocampus, Range = 2
**Substantia nigra**				
−12	−21	−6	5.259	Left Brainstem, Midbrain, Gray Matter, Substania Nigra, Range = 1
**Uncus**				
21	6	−18	4.642	Right Cerebrum, Limbic Lobe, Uncus, Gray Matter, Brodmann area 28, Range = 0
21	−6	−21	5.159	Right Cerebrum, Limbic Lobe, Uncus, Gray Matter, Amygdala, Range = 0
**Putamen**				
−30	−9	−6	4.770	Left Cerebrum, Sub-lobar, Lentiform Nucleus, Gray Matter, Putamen, Range = 1
**Claustrum**				
36	6	−6	5.304	Gray Matter nearest to (36, 6, −6): Right Cerebrum, Sub-lobar, Claustrum, Gray Matter, Range = 3
**CEREBELLUM**				
3	−30	−18	5.456	Right Cerebellum, Anterior Lobe, Culmen, Gray Matter, Range = 5
24	−63	−18	5.778	Right Cerebellum, Posterior Lobe, Declive, Gray Matter, Range = 0
18	−90	−18	4.137	Right Cerebellum, Posterior Lobe, Declive, Gray Matter, Range = 2
6	−39	−21	5.193	Right Cerebellum, Anterior Lobe, Culmen, Gray Matter, Range = 0
9	−45	−21	5.004	Right Cerebellum, Anterior Lobe, Culmen, Gray Matter, Range = 0
12	−63	−21	5.759	Right Cerebellum, Posterior Lobe, Declive, Gray Matter, Range = 0
12	−84	−21	3.768	Right Cerebellum, Posterior Lobe, Declive, Gray Matter, Range = 0
−9	−48	−3	4.514	Left Cerebellum, Anterior Lobe, Culmen, Gray Matter, Range = 0
−12	−54	−6	4.736	Left Cerebellum, Anterior Lobe, Culmen, Gray Matter, Range = 0
−33	−72	−15	6.062	Left Cerebellum, Posterior Lobe, Declive, Gray Matter, Range = 0
−9	−36	−18	4.405	Left Cerebellum, Anterior Lobe, Culmen, Gray Matter, Range = 0
−3	−78	−18	5.505	Left Cerebellum, Gray Matter, Range = 0
**BRAINSTEM**				
9	−21	−3	4.800	Right Brainstem, Midbrain, Gray Matter, Red Nucleus, Range = 0

### Audio-visual perception and attention

During task execution in the scanner, sensorial perception and word recognition occur. We found related bilateral neural activity in the thalamus, which is known to process incoming information and to relay it to related specific areas in the cortex (Mitchell et al., 2014). Further, auditory cortices BA 38 along with BA 41 and BA 42 (the transverse temporal areas) responded upon acoustic word perception. In addition to processing auditory information, these areas also access stored representations of words (Scott and Wise, 2004). Other regions were engaged in the task: BA 22 maps sound and word meaning (Zhuang et al., 2014), the left fusiform gyrus BA 37 is implicated in reading (McCandliss et al., 2003), and the supramarginal gyrus BA 40 mediates word recognition (Stoeckel et al., 2009; Wilson et al., 2011). Also the frontal eye field BA 8 was active and possibly engaged in attention processes (Esterman et al., 2015).

### Lexical recognition

Zhuang et al. (2014) describe lexical recognition as consisting of two related processes: competition and selection. In competition, cohort candidates, i.e., words sharing some phonological feature(s) with the word presented compete with each other for a match. These cohort words are stored in memory areas that become active upon search. We found activity in an extended memory network engaged in word competition, including the right hippocampus (Smith et al., 2011; Huijgen and Samson, 2015), the para-hippocampal gyrus, BA 27, BA 36 bilaterally (Squire and Dede, 2015), and the left temporal lobe BA 38 (St. Jacques et al., 2011). In our data, the process of selection involved the left inferior frontal gyrus and also its right counterpart, BA 44, 46, the left BA 47, cortical regions found previously during the completion of this task (Heim et al., 2005; Rodd et al., 2012; Zhuang et al., 2014), and the insula, which is also engaged in word processing (Zaccarella and Friederici, 2015). Contrary to this last study, we did not find involvement of the pars triangularis of the inferior frontal gyrus BA 45. Instead, we detected activity in BA 9, a region known to be active in working memory mediating selection tasks, in interplay with BA10, and in the anterior cingulate cortex BA 32 and BA 34 (Zhang et al., 2003), areas also present in our network.

### Cognitive control

Our results suggest that mechanisms of cognitive control might also contribute to the network engaged in word recognition. We found increased activity in the anterior cingulate cortex BA 31 and 34 as well as in its posterior portion BA 31, in the middle and superior frontal gyri BA 9, and in the frontopolar prefrontal cortex BA 10. Previous studies bundled these regions into a network that detects input diverging from a stored template and suppressing it (Botvinick et al., 2001; Cole and Schneider, 2007). Hence cognitive control could account for adequate item selection, suppression of cohort candidates, and novel words presented during the task. However, our analysis does not allow discernment of whether the cingulate cortices and BA 10 contribute to selection within the network for cognitive control or whether selection processes and dedicated networks include the network of cognitive control.

### Experience-related sensorimotor word networks

Our investigation aimed to detect how words learned with iconic gestures are functionally mapped into neural tissue. In addition to the core language network (Friederici, 2011) described in the preceding section we found a number of premotor, motor, and sensorimotor areas that were activated during word recognition. Most remarkably, upon audio-visual presentation of the words (note that no videos of the actress performing gestures were shown during the scanning procedure), the brain images unveiled activity in large portions of the left premotor cortex BA 6, which is engaged in movement preparation and simulation. The right counterpart was minimally involved. We attribute this imbalance to the fact that all subjects were right-handed (Tettamanti et al., 2005). Activity in the premotor cortices as preparation of words referring to motor acts is well documented in numerous neuroimaging studies in which words were presented either visually or acoustically or both (Hauk et al., 2004; Pulvermuller, 2005; D'Ausilio et al., 2009; Cappa and Pulvermüller, 2012; Berent et al., 2015). In our study, the pattern of response to audio-visual word presentation also involved the left primary motor cortex, where BA 4 and BA 7 play a role in motor sequence coordination, visuo-motor coordination, planning of complex movements, and proprioception (Baker et al., 2012). Additionally, our data revealed involvement of the basal ganglia, i.e., left putamen, left and right caudate, left substantia nigra, and the cerebellum, which bilaterally contribute to motor emulation processes (Lotze and Halsband, 2006; Ridderinkhof and Brass, 2015). We ascribed the engagement of the motor regions during word recognition to the experience collected by our subjects while learning. In retrieval, motor acts are unconsciously simulated as associated to the phoneme and letter sequence(s). Similarly, primary somatosensory cortices BA 3 and BA 5, also present in our analysis, converge to create a proprioceptive trace within the network related to gesture execution (Pleger and Villringer, 2013). Perception of space and body location mediated by the supramarginal gyrus BA 40 and the angular gyrus BA 39 are also part of a word's representation (Blanke, 2012). Besides involvement of sensorimotor areas, activity in BA 18, a visual association area, reflects the complex image processing that occurs during training and the reactivation of a mental image (Lambert et al., 2004). Subjects were cued to read the words and watch the videos of an actress performing the iconic gestures. Involvement of the right fusiform gyrus might thus mirror the input, i.e., the actress' face (Morris et al., 2007) and body (Soria Bauser and Suchan, 2015) might also be mapped into the word's representation. Altogether, these data reflect the sensorimotor input processed during learning. The contrast images provide evidence of word learning as a cognitive process grounded in embodied experiences (Pulvermuller, 1999).

## Discussion

The present study is explorative. Also, this fMRI-data analysis has a limitation: it cannot disentangle the different processes that occurred in the scanner, i.e., word perception, recognition, and the functional neural mapping of the words created through sensorimotor experiences. Brain areas engaged in multiple functions partially overlap in the different processes, i.e., sensorial processing, the recognition task and sensorimotor emulation of the words.

However, the data provide insight into the functional representation of words in a foreign language learned through iconic gestures. The results support Engelkamp's and Krummnacker's seminal theory on enactment (1980), which proposed that performing a gesture when memorizing a word leaves a motor trace in the word's representation. Furthermore, our results can be embedded in theories of embodied language that have emerged in the past decade. Based on neuroscientific evidence, these studies maintain that language (along with other processes in cognition) is grounded in bodily experiences created with our sensorimotor systems (Barsalou, 1999, 2008; Pulvermuller, 1999; Gallese and Lakoff, 2005; Fischer and Zwaan, 2008; Jirak et al., 2010; Glenberg and Gallese, 2012). When seeing a fruit basket, children get to grasp a fruit. Holding the fruit, children smell it, put it in their mouth, taste it, drop it, pick it up, squeeze it, and feel the pulp and dripping juice; while pointing to the fruit while caregivers produce a sequence of sounds [ˈɒr.ɪndʒ], *orange*, the “concept's name” (Oldfield and Wingfield, 1965). Connecting all sensorimotor experiences with the sound, children try to reproduce it. By doing so, children combine multiple bodily experiences related to the fruit and create an embodied concept connected to a sound string that becomes a part of this representation. One day children will learn to write the sound sequence. At that point, children acquire another system—graphemes—in order to label the concept and to communicate about these experiences in a written way. Spoken and written words thus become a component of the concept, and as such they become connected with the embodied concept itself. Considering this, words are not abstract units of the mind (Fodor, 1976, 1987). Instead, words are grounded to a great amount in bodily experiences (Gallese and Lakoff, 2005). A number of studies conducted over the past decade have demonstrated the involvement of the body in conceptual representations. Most of these studies have used written words that participants read silently in the fMRI scanner. Reading action words activated motor cortices (Kemmerer et al., 2008; Cappa and Pulvermüller, 2012). This process occurred selectively depending on the effector of the body involved in the action (Carota et al., 2012). Action words (such as *kick, pick*, and *lick*) that refer to actions performed with leg, arm or mouth elicited activity in regions of the motor cortex controlling their movement (Hauk et al., 2004). González et al. (2006) made participants read odor words and found brain activity in regions that are not related only to the task, i.e., canonical language areas involved in reading, but more interestingly in olfactory brain regions. Note that participants had no perception of odor. Similarly, mere reading of gustatory words such as *salt* engaged gustatory regions in the brain (Barrós-Loscertales et al., 2012).

In recent years, embodiment research has shown that a good portion of abstract words are also grounded in bodily experiences. In the fMRI scanner, Moseley et al. (2012) had participants read abstract emotion words such as *fear, dread*, and *spite*. In the subjects' brains, the researchers detected activity in emotional networks, but more interestingly in those portions of the premotor cortex engaged in movement preparation for arm- and face-related gestures. Such gestures ground the social expression of feelings. Anecdotally speaking, if asked to demonstrate the concept of grief, in Western culture we might produce a certain facial expression or mime wiping our eyes to indicate crying. Hence even if, linguistically, the word is abstract, our interpretation of it is embodied. We have learned the spoken or written word for a concept that is not abstract but related to emotional and bodily experiences.

Additionally, metaphors involving body parts also are reported to evoke embodied reactions in the brain. Boulenger et al. (2009) asked whether somatotopic responses in the motor cortex occur during reading of metaphoric sentences such as “John grasped the idea” compared to literal sentences such as “John grasped the object.” The results showed activity in motor cortices for both metaphoric and literal sentences. Another study (with magnetic encephalography) produced similar results (Boulenger et al., 2012). Lacey et al. (2012) had participants read sentences such as “She has steel nerves.” and “Life is a bumpy road.” that contained texture metaphors, along with control sentences such as “She is very calm.” and “Life is a challenging road.” Texture metaphors induced activity in the somatosensory cortex and more specifically in texture-selective areas. Altogether, the reviewed studies suggest that interaction with the world creates brain topographies of concepts and reflects a word's semantics (Pulvermüller, 2002; Moseley and Pulvermüller, 2014).

Our data do not overtly support the hypothesis on enactment that attributes memory enhancement to visual imagery (Backman et al., 1993; Knudsen, 2007; Muzzio et al., 2009a,b). However, it stands to reason that the word network also comprises a mental image of the gesture's execution from a first-person perspective; hence the preparation to perform the gesture with the premotor cortex, supplementary motor area (Park et al., 2015), motor cortices, basal ganglia, and cerebellum are involved. However, the word network might also comprise a kinetic mental image from a second-person perspective, i.e., the subject's unconscious rehearsal of the actress during gesture performance. This perspective could be mapped in the higher visual association areas, the fusiform gyrus, the supramarginal, and the angular gyrus as described in the results section of this paper. Other regions, e.g., hippocampus, posterior cingulate cortex, medial prefrontal cortex, and angular gyrus, identified in imagery also are contained in the activated network and found in other studies (Huijbers et al., 2011; Ridderinkhof and Brass, 2015).

### Network complexity and memory

Our results confirm one of the theories on enactment asserting that complexity of word representation determines memory enhancement (Knopf, 1992; Kormi-Nouri, 1995; Macedonia, 2003). Theoretically, this position is embedded in connectionist models of memory. In connectionism, a concept can be described by means of networks representing and storing information (McClelland, 1985; McClelland and Rumelhart, 1985; McClelland and Rogers, 2003). Concept networks consist of nodes and edges. Nodes can be seen not only on an abstract level but also in a more biological understanding as neural assemblies and cortical areas wiring together on different scales upon synchronous stimulus processing (Douglas and Martin, 2004; Singer, 2013). During learning, synchronous firing of neurons (*coincident activation*) leads to changes in the weight of connections (Hebb, 1949) among neurons. Through these changes in connections, neurons (re)organize in functional units in different dimensions that process and store information. According to connectionist models, concepts are not stored locally in the brain. Instead, concepts are represented in a distributed way (Lashley, 1950). Thus, a concept is not considered as a single unit but as a pattern clustering different components wired together during learning. A growing body of evidence has demonstrated this position in neuroscientific studies (for a review, see Pulvermüller, 2013). In connectionist networks, word retrieval is driven by spreading activation within the network. Activation starts at one or more nodes, depending on the input, and triggers activity in the whole network that ends after the search has been completed by inhibition (McClelland, 1985). The classic example described by McClelland et al. (1986) is the rose. The smell and the appearance of the flower are experienced synchronously. After a certain number of experiences, the two components become interconnected. Visual processing of a rose will activate the network. Hence the node storing the smell will be reached by activity and the smell sensation will be triggered even in absence of a real rose. Twenty years later, González et al. fMRI-study (González et al., 2006) connected neuroscientific evidence to McClelland's rose example.

One basic assumption in neural networks theories is that complexity in representation makes memories stable and longer lasting (Klimesch, 1994). The more nodes a concept has, the more stable the concept's representation is. In fact, if a node within the network decays, activity in the network can be started from other nodes and information can be restored. For example, a word in L2 that has been learned with a picture will be more complex in its representation than a word that has been learned in its written form. This will enhance the word's memorability, as shown in a recent study by Takashima et al. (2014). Paradoxically, if a word network in L2 consists of only one node, for example a string of sounds in a foreign language, decay could affect it fatally. A gesture accompanying a word engages many brain regions and therefore provides a complex representation of the word. It enriches the sound or character string with sensorimotor information, it makes its representation complex, and it enhances retrievability compared to audio-visual learning only. This is the case in both the short (Macedonia and Knösche, 2011) and the long term (Macedonia and Klimesch, 2014) when the network is impacted by decay.

### Procedural memory for words learned with iconic gestures

Our data also allow a further interpretation. Considering the high involvement of the motor system in the word recognition task, as described in the Results Section, it becomes plausible to assume that procedural memory might be engaged in word learning. Procedural memory is implicit, long-term, and grounded in the motor system when a person acquires a skill. In cognitive science, vocabulary theoretically is situated in the domain of declarative memory (Tulving and Madigan, 1970; Ullman, 2004; Cabeza and Moscovitch, 2013; Squire and Dede, 2015). However, because of the procedure used to acquire the words (iconic gestures accompanying the words, i.e., well defined motor acts/programs), it stands to reason that both declarative and procedural memory systems might interact and jointly accomplish word storage and retrieval. In activation Table 1, we find brain regions typically mediating declarative memory (Nikolin et al., 2015), i.e., hippocampus, para-hippocampal (Nadel and Hardt, 2011), and the fusiform gyri (Ofen et al., 2007), as well as regions within the prefrontal cortex, the dorsolateral prefrontal cortex (Blumenfeld and Ranganath, 2007), and the medial temporal lobe (Mayes et al., 2007). This provides evidence that words learned during our experiment are stored in declarative memory. At the same time, our activation list reports brain regions mediating procedural memory, in addition to (pre)motor regions, the basal ganglia (Barnes et al., 2005; Yin and Knowlton, 2006; Wilkinson and Jahanshahi, 2007) and the cerebellum (D'Angelo, 2014). Thus, word learning, if accompanied by gestures seems to recruit both memory systems. This might be responsible for superior memory performance in storage and retrieval of verbal information. Our results are in line with literature considering declarative and procedural memory as interacting as opposed to being distinct (Davis and Gaskell, 2009), and with a more recent review of patient and animal studies indicating that the medial temporal lobe and basal ganglia mediate declarative and procedural learning, respectively, depending on task demands (Wilkinson and Jahanshahi, 2015).

## Conclusion

In this paper, we were interested in the functional neural representation of novel words learned with iconic gestures. Our study is explorative and one of our aims was to detect brain regions that play a special role in memory enhancement. Besides networks engaged in attention and word recognition, we found a word network that maps the words according to the modalities engaged in the learning process. We attribute the superior memory results induced by gestures to the complexity of the network. Complex sensorimotor networks for words store verbal information in an extended way, conferring stability on the word's representation. Our brain data also shows that learning words with gestures possibly engages both declarative and procedural memory. The involvement of both memory systems thus explains why learning is enhanced and information decay is delayed as shown in behavioral long-term studies. The implications for education are clear: gestures should be used in second language lessons in order to enhance vocabulary learning.

## Author contributions

MM Experimental design, stimuli creation and recording, data acquisition and analysis, paper writing. KM Supervision in data acquisition and analysis.

### Conflict of interest statement

The authors declare that the research was conducted in the absence of any commercial or financial relationships that could be construed as a potential conflict of interest.
